# Fever-Associated Seizures or Epilepsy: An Overview of Old and Recent Literature Acquisitions

**DOI:** 10.3389/fped.2022.858945

**Published:** 2022-04-21

**Authors:** Piero Pavone, Xena Giada Pappalardo, Enrico Parano, Raffaele Falsaperla, Simona Domenica Marino, John Kane Fink, Martino Ruggieri

**Affiliations:** ^1^Unit of Clinical Pediatrics, AOU “Policlinico”, PO “G. Rodolico”, University of Catania, Catania, Italy; ^2^Unit of Catania, National Council of Research, Institute for Research and Biomedical Innovation (IRIB), Catania, Italy; ^3^Department of Biomedical and Biotechnological Sciences (BIOMETEC), University of Catania, Catania, Italy; ^4^Unit of Pediatrics, Neonatology and Neonatal Intensive Care, and Pediatric Emergency, AOU “Policlinico”, PO “San Marco”, University of Catania, Catania, Italy; ^5^Department of Neurology and Ann Arbor Veterans Affairs Medical Center, University of Michigan, Ann Arbor, MI, United States; ^6^Unit of Rare Diseases of the Nervous System in Childhood, Department of Clinical and Experimental Medicine, Section of Pediatrics and Child Neuropsychiatry, University of Catania, AOU “Policlinico”, PO “G. Rodolico”, Catania, Italy

**Keywords:** febrile seizures (FS), febrile status epilepticus (FSE), febrile infection-related epilepsy syndrome (FIRES), Dravet syndrome (DS), genetic epilepsy with FS plus (GEFS+), new-onset refractory status epilepticus (NORSE), fever-associated seizures or epilepsy (FASE)

## Abstract

In addition to central nervous system infections, seizures and fever may occur together in several neurological disorders. Formerly, based on the clinical features and prognostic evolution, the co-association of seizure and fever included classical febrile seizures (FS) divided into simple, complex, and prolonged FS (also called febrile status epilepticus). Later, this group of disorders has been progressively indicated, with a more inclusive term, as “fever-associated seizures or epilepsy” (FASE) that encompasses: (a) FS divided into simple, complex, and prolonged FS; (b) FS plus; (c) severe myoclonic epilepsy in infancy (Dravet syndrome); (d) genetic epilepsy with FS plus; and (e) febrile infection-related epilepsy syndrome (FIRES). Among the FASE disorders, simple FS, the most common and benign condition, is rarely associated with subsequent epileptic seizures. The correlation of FS with epilepsy and other neurological disorders is highly variable. The pathogenesis of FASE is unclear but immunological and genetic factors play a relevant role and the disorders belonging to the FASE group show to have an underlying common clinical, immunological, and genetic pathway. In this study, we have reviewed and analyzed the clinical data of each of the heterogeneous group of disorders belonging to FASE.

## Introduction

Several disorders are characterized by the co-occurrence of fever and seizures. Among these, central nervous system (CNS) infections, including meningitis, encephalitis, and brain abscesses, are often associated with seizures in children. A correct differential diagnosis from febrile seizures (FS) leads to an appropriate treatment, possibly preventing further severe complications. With the exclusion of infectious agents in the brain, the association of seizures with fever was recognized as FS, divided into simple and complex, based on clinical features and possible evolution to epilepsy and other neurological complications (1). The correlation between FS and epilepsy has been widely documented (1–5). In fact, Deng et al. (3) introduced the term “fever-associated seizures or epilepsy” (FASE) as a disorder primarily characterized by the occurrence of a seizure or epilepsy usually accompanied by fever. According to Deng et al. (3), FASE includes the following disorders: (a) simple, complex, and prolonged (febrile status epilepticus) FS (4, 5); (b) febrile seizures plus (FS+) (6); (c) severe myoclonic epilepsy in infancy (Dravet syndrome (DS) (7); (d) genetic epilepsy with febrile seizures plus (GEFS+) (8); and (e) febrile infection-related epilepsy syndrome (FIRES) (9, 10). More recently, Ding et al. (11) have conducted in a study aimed to identify disease-causing gene mutations in individuals with FASE diagnosis. Here, we reviewed and analyzed the clinical data of each component of FASE group's disorders as they share an underlying common clinical, immunological and genetic pathway.

## Fever-Associated Seizures Or Epilepsy (FASE)

In children with FASE, fever and seizures are the primary features; however, clinical assessment, type of treatment, and prognostic evaluation are widely different for each of these disorders, such as simple FS with short-duration seizure and no complication in contrast to complex FS, and in particular, with prolonged FS, which possibly lead to severe neurological implications (12, 13). Family history, normal milestone acquisitions, brief resolution of the seizure event, and rapid regain of consciousness are signs indicative of a benign course. Certainly, the first occurrence of seizures in the presence of fever will prompt complete clinical evaluation to exclude CNS infection. The clinical course of the previous FS may help to exclude further disorders of the FASE group. The etiology of FASE is poorly known but, however, is thought to be multifactorial caused by: increased neuronal vulnerability of the child during the growth affected by a low threshold for seizures; fever as a trigger factor; and, familial genetic predisposition, as shown by clinical concordance with one of the parents (30%), and with monochorionic twins (35–69%) (14, 15). An increased risk of FS has also been observed in children with iron deficiency and low levels of other micronutrients, such as vitamin B12, folic acid, selenium, calcium, and magnesium (16). Mosili et al. (17) sustain that some cytokines (interleukins and tumor necrosis factor) play an important role in altering the permeability of the blood-brain barrier. The passage of cytokines into the CNS induces the fever through the hypothalamic stimulation on one hand, and inducing seizures through the glutamatergic and GABAergic dysfunction on the other hand. Also, the role of genetic mutations in the pathogenetic mechanism of FASE is relevant, as demonstrated by the fact that the same mutation may express different phenotypes. Instead, with regard to the action of fever stimulating the seizure, Deng et al. (3) hypothesized that high temperature may stimulate the recycling of synaptic vesicle and enlarge the size of synaptic vesicle with subsequent enhancement of synaptic transmission and onset of seizures. As previously reported, FASE comprises common and well-known disorders, such as FS; however, new scenarios, particularly in the field of clinical presentation, genetics, and immunology, have been evolving, expanding the boundaries of these disorders, particularly regarding FIRES and related disorders.

### Febrile Seizures (FSs)

FSs are the most common seizure events in children aged <5 years (1, 4, 13) with frequencies of 2.3–2.7% in the United Kingdom (4). In general, in recent years, admission of children with FS to the emergency room has been notably reduced in more developed countries because of the wide use of buccal midazolam or rectal benzodiazepine administered directly by the parents at home. Viral infections involving the ear, nose, throat, and upper respiratory tract are the most frequent causative events of fever-associated seizures. In a retrospective study, Tarhani et al. (18) have reported on 77 children affected by FS that in these children the mean duration of seizures was 5.09 +/– 3.78 minutes and the mean temperature during seizures was 38.41 +/– 0.83 °C. In 44 (57.14%) children no cause of the fever was documented. Ten (12.99%) children had multiple seizures within 24 h, in 70 (90.91%) the seizures ended without medication and 5 (6.49%) were treated with diazepam.

Stokes et al. (19), in a cohort of 276 children with FS identified by the viral analysis of nasopharyngeal secretions and cough/nasal swabs, found viral infections in 49% of the children with influenza A, respiratory syncytial virus (RSV), and adenoviruses, being the most frequent causes of infections. Norovirus gastroenteritis has been reported as a causative event in children with febrile and afebrile seizures. Among 108 children affected by norovirus gastroenteritis and seizures, 49 (45.4%) were diagnosed with FS, whereas 59 (54.6%) exhibited afebrile seizures (20). Wang et al. (21) in a cohort of 868 children with FS associated with mild gastroenteritis showed that 67 (7.7%) had a second afebrile seizure (AS) and 71% (48/67) showed gastroenteritis-associated recurrence. According to these authors, the risk of AS relapse after the initial mild gastroenteritis is low.

FSs are classified as simple (typical), complex (atypical) (5, 22, 23), or prolonged (febrile status epilepticus) based on the temperature level, mode of onset, duration of the crisis, recurrence, seizure semiology including post-ictal event, and the occurrence of interictal neurologic signs (22–24).

### Febrile Seizures: Simple (Typical) vs. Complex (Atypical)

The main characteristics of the simple, complex, and prolonged FS are reported in Table 1. Simple FS is defined as a short (<15 min) generalized seizure, not recurring within 24 h, occurring during a febrile illness not caused by an acute disease of the nervous system in a child aged between 6 months and 5 years, without neurologic deficits and with no previous afebrile seizures. As experienced by the authors of the article, and also as reported in the literature, the onset of typical FS before the age of 7 months is quite uncommon. The onset occurs more frequently before the age of 3 years, with a peak between 18 and 24 months. Seizures tend to appear during the phase of the rising fever. Seizures typically have a short duration, presenting as generalized tonic-clonic without post-ictal paralysis. There were no other seizure recurrences in the same febrile episode. Almost all children with simple FS have a good prognosis for both normal neurologic function and the occurrence of epileptic seizures (4–6, 21–24). Although the prognosis for subsequent epileptic seizures in children with simple FS is similar to that of the general population, the risk of afebrile seizures is increased to 15–20% among children with complex FS (5, 13). Clinical events may simulate FS, including muscle tremors, acute confusional state/delirium, and febrile myoclonus. This latter brief presents with involuntary muscle contractions and myoclonic movements primarily affecting the lower limbs, temporally related to febrile episodes or following infectious illnesses (25, 26). In evaluating a child with FS, it is important to (i) obtain a family history particularly focused on neurological disorders; (ii) conduct a cautious physical examination to exclude direct CNS infections; and, (iii) carry out detailed laboratory analysis, including full blood count, electrolytes, and glucose. Family history of simple FS, brief duration of the seizure, and rapid regain of consciousness are highly indicative of the diagnosis, preluding further investigations, such as electroencephalogram (EEG) recording, lumbar puncture (LP), and brain magnetic resonance imaging (MRI) (27, 28). FS that does not meet the criteria of the simple type is classified as complex FS. Complex (atypical) FS is often focal and may be accompanied by post-ictal paralysis. In contrast to simple FS, complex FS is of longer duration and may recur during the same febrile episode. The need for LP in patients with simple or complex FS is debatable. Batra et al. (27) maintain that in children with fever and seizures, the likelihood of meningitis diagnosis is low. In their study, in a cohort of 497 children aged 6–18 months admitted to the emergency room with a first FS, 1 (0.8%) of 116 children with simple FS and 4 (5%) of 63 children with complex FS were diagnosed with meningitis (26). Similarly, Kimia et al. (28) noticed that the risk of bacterial meningitis in children aged 6–18 months presenting with first simple FS was low. Clearly, the option for LP in simple and complex FS children must be carefully evaluated because a missed diagnosis of intracranial infection may become the cause of severe complications. LP is considered unnecessary in children with simple and complex FS who have returned rapidly to a normal baseline after the seizure and in the absence of symptoms or signs of cerebrospinal infection (23). According to the American Academy of Pediatrics, LP should be limited to infants with FS who did not receive *Haemophilus influenzae* type b (Hib) or pneumococcal vaccines and those under antibiotic treatments shortly or during the current illness (29). FS may reoccur in approximately one-third of children with FS, generally within 12 months following the initial FS. Approximately half of the children with a second FS will have a third FS. High recurrence of simple FS is not commonly reported (2).

**Table 1:** Differences between simple (typical), complex (atypical), and prolonged febrile seizures.

	**FS simple**	**FS complex**	**Prolonged**
Onset	During the rising phase of highly febrile episodes	Low or moderate level of febrile episodes	Continuous/intermittent febrile seizures without consciousness being regained at the interictal state for more than 30 mins
Duration	Few minutes	More prolonged (10 mins)	
Phenotype	Generalized tonic-clonic	Hemilateral or focal	
Recurrence during the same fever episode	No recurrence	Possible recurrence	
Others FS	Frequent	More frequent	
Post-ictal events	No	May be residual Tod's paralysis	
Prior neurologic signs	Absent	May be present	
Prognosis	Good as the general population	Afebrile seizures in 10–15% of cases	

In the group of children with initial FS complex, the risk of subsequent afebrile seizures increases, reaching 15–20% in association with one or more of the following factors: developmental delay, previous neurological dysfunction, cerebral palsy, family history of epilepsy, and recurrence within the same infectious episode (5). The relationship between complex FS and the subsequent onset of epileptic seizures is unclear. Previous cerebral damage may be a cause for both disorders; most accepted is the hypothesis of an interaction of predisposing factors acting for both disorders at a different time. High FS recurrence is linked not only to the risk of subsequent epilepsy (17), but may also be correlated with cognitive and language delay (30), Tourette syndrome (31), and attention deficit hyperactivity disorder (32). In a study published in 2004, Pavone (23) reported clinical features of the cases seen in his institution from 1996 to 2004 that may be included as an example into FASE syndrome classification.

### Vaccination and Acute Febrile Seizures Treatment

The first FS episode is highly traumatic and frightening for the parents and may cause great anxiety for each new febrile event. It is important to advise parents to avoid contact between children and relatives and schoolfellows affected by a contagious disease. Vaccination is another issue of concern for the parents. Children with FS may develop a fever after regular vaccination; however, the risk of severe complications is considered low. In cases of fever after revaccination, antipyretics and anticonvulsants may be administered at home. The term vaccine proximate seizures (VPS) defines episodes of seizures as febrile or afebrile occurring 14 days after vaccination. In a retrospective study conducted by Deng et al. (33) on 119 children with VPS as the first seizure episode, the chance of further seizures with revaccination was uncommon. According to these authors (33), in children who had a single VPS as their only seizure, the recurrence of VPS was uncommon. Children who had multiple non-vaccine proximate seizures after their initial VPS (VPS+) were more likely to have afebrile VPS at a younger age and have a VPS recurrence with vaccination. In the context of VPS, cases of “afebrile benign convulsion” may occur, and the causative event is not directly linked to fever. In this case, it is not the fever that is pathogenetic, but the inflammation due to the agent causing the underlying inflammatory diseases similar to what happens with norovirus gastroenteritis (20). Acute treatment of simple and complex FS after VPS is symptomatic and directly related to the causal event of fever using antipyretics and appropriate drugs. In our institution, in the emergency room, acute treatment of FS consists of intravenous administration of diazepam (0.1–0.2 mg/kg) or lorazepam (0.05–0.1 mg/kg). Oral, rectal, or intranasal treatment of benzodiazepines may be used directly by the parents at home before visiting the hospital as commonly recommended (29, 34).

## Prolonged Febrile Seizures (Febrile Status Epilepticus)

Most of the FS are of brief duration and stop after a few minutes without treatment. In 9% of cases, FS is of long duration, and among these, 5.8% meet the criteria for febrile status epilepticus (FSE) (35, 36). FSE is defined as a continuous or intermittent febrile seizure without consciousness being regained in the interictal state for more than 30 min (35, 37). Recently, the limit of 30 min has been slightly lowered (24, 28). In prolonged febrile seizures, LP and cerebrospinal fluid examination for looking at pressure, cell count, protein, and glucose levels are necessary to exclude a cerebrospinal infection. In a study including 756 children aged 6–60 months, 532 had simple FS, 194 had complex FS, and 39 had FSE (35). A significant relationship was found between FSE and complex FS with respect to body temperature during a seizure, family history of FS, and premature birth (36). Likewise to other types of FS, viral infections have been implicated as the main cause of FSE. Hall et al. (38) reported that primary and reactivated human herpes virus 7 (HHV-7) infections showed similar clinical manifestations, except for seizures manifesting more frequently in reactivated infections and influenced by the interaction of HHV-7 and human herpes virus 6 (HHV-6). Uda and Kitazawa (39) conducted a study on 99 children with FSE aged <3 years who had been tested for RSV. Three patients in the RSV-positive group (*n* = 19) and four in the RSV-negative group (*n* = 80) exhibited bronchiolitis, and six children in the RSV-positive group developed encephalopathy and profound neurological sequelae. The authors maintain that RSV infection in the absence of bronchiolitis can initially manifest with FSE and subsequently develop into acute encephalopathy. In a prospective multicenter study regarding the consequences of prolonged febrile seizures (FEBSTAT-study) (40), 169 children aged from 1 month to 5 years were evaluated for HHV-6 and HHV-7 infections. Specific viral infections are linked to the onset of FSE; HHV-6 infections are commonly associated with the onset of FSE, whereas HHV-7 FSE has been reported less frequently (40). Together, these infections encompass one-third of the cases of FSE. These authors establish that no clinical differences are observed in children with FSE presenting with or without HHV infection (40).

FSE has been associated with an increased risk of subsequent FSE and further neurological complications, including epilepsy and cognitive disability (41). The relationship between FSE and hippocampal sclerosis and mesial temporal lobe epilepsy (MTLE-HT) has long been debated (42). It has been assumed that FS at an early age may affect the hippocampus, and, subsequently, causing FSE and MTLE-HT (35, 36). Additional hypotheses refer to previous hippocampal damage because of pre-perinatal or predisposing genetic factors, or to the anatomic hippocampal anomaly that individually or in association may result in both prolonged FS and MTLE-HT. Lewis et al. (41) found hippocampal T2 hyperintensity in association with increased volume in 22 out of 226 children after FSE, thus showing an acute hippocampal injury that was often found to evolve to hippocampal sclerosis after 1 year. Opposite results were obtained by Tarkka et al. (42) in a study of 24 patients with prolonged first FS. They compared 8 children with an unprovoked seizure after the first FS with 32 patients as the control group; none of the patients had mesial temporal sclerosis. FSE is generally focal, occurs in very young children, and generally appears as the first FS (36). FSE is fairly resistant to drugs; however, early treatment from seizure onset results in a shorter seizure duration (36, 43). One study has reported that although FSE does not appear to be associated with significant cognitive impairment in the short term, it may result in a trend for possible receptive language and motor delay a year after FSE onset (44). Myers et al. (45) reported a 20-month-old girl with a complex chromosomal disorder who died suddenly 2 days after FSE, suggestive of sudden unexplained death in epilepsy (SUDEP). Although seizures are a notable risk factor for SUDEP, the mechanism by which seizures are the cause of death remains unclear. Nei and Hays (46) maintained that seizures activate specific cortical and subcortical regions that can cause potentially lethal cardiorespiratory changes. The role of brain malformations observed in this condition remains unclear (47). Protocadherin-19 mutations may present with developmental encephalopathy and prominent infantile-onset focal seizures that show limbic abnormalities in a quantitative MRI-based analysis (48). Results of serum growth and differentiation factor (GDF)-15 has been shown to be a potential prognostic biomarker for patients affected by febrile status epilepticus (49).

## Febrile Seizures Plus (FS+)

Scheffer and Berkovic (6) initially used the term FS plus (FS+) to describe the syndrome with multiple FS (onset at a median age of 1 year) persisting over the age of 6 years and presenting with febrile or afebrile generalized tonic-clonic seizures. More recently, designation of FS+ has been applied to individuals with onset of FS before 3 months or after 6 years of age and occurrence of both febrile and afebrile generalized tonic-clonic seizures either limited to the usual age for FS (3 months to 6 years) or occurring outside this period (50).

## Dravet Syndrome (Ds)—Severe Myoclonic Seizures In Infancy

The first description of this syndrome was reported by Dravet in 1978, who subsequently described the classical clinical features of severe myoclonic seizures with onset in infancy, associated with frequent episodes of status epilepticus, often triggered by fever. The syndrome was first described as severe myoclonic seizures in infants and was associated with various types of severe epileptic seizures (7, 51). In classical clinical expression, the disorder starts with the onset of FS before the age of 1 year in a child with normal growth and developmental stages. The temperature is generally mild or moderate, although it may sometimes be elevated. The febrile-associated seizures may be generalized or focal. In most cases, aside from the precocious onset of seizures at 6–8 months, no other indicative diagnostic signs are present. On some occasions, the seizures may manifest without fever. Brain MRI and EEG initially yield normal results. In the subsequent years, multiple seizure types, mainly myoclonic, atypical absence, and focal seizures, have been reported with progressive slowing of developmental and cognitive skills (52). Five principal diagnostic criteria for DS have been indicated: normal development before seizure onset; two or more FS complexes with onset before 12 months; phenotypic aspect of seizures, which may be focal myoclonic, hemiclonic, or generalized tonic-clonic; two long-duration seizures; and refractory seizures after the age of 2 years (7, 52).

A consensus building, that is, a modified Delph process, was conducted to indicate the standards for early, long-lasting, effective, and accurate diagnosis of DS (52). The other reasons for the consensus regarded optimal therapies for seizures, and recommendations for evaluation and management of the conditions for children and adults affected by the syndrome (52). The main characteristics of the clinical presentation obtained by this consensus were: typical onset between 1 and 18 months; recurrent generalized tonic-clonic or hemilateral seizures (considered as diagnostic), often prolonged; occasionally brief in duration; myoclonic seizures by the age of 2 years; blunting status; focal dyscognitive seizures; atypical absence after the age of 2 years; atypical epileptic spasms; seizures triggered by hyperthermia; and normal developmental and neurological examination at the onset with normal EEG and brain MRI (52). DS may have various clinical manifestations with different ages of presentation, type of seizures, and absence of cognitive impairment (53). DS may involve other structures aside from the brain with cardiac, hearing, vision, urinary, bowel, and endocrine dysfunctions (54–56). The syndrome is uncommon, with a reported incidence of 1 in 15.700 to 14 in 40.900 (57). Li et al. (58) reported on 205 patients with DS with a median age of 8.5 years. Twenty-five patients were deceased. In this study, the authors underlined the following clinical features of DS: the first seizure is febrile in only 55% of patients, and 3% of patients never have seizures with fever; more than 50% of patients manifest with a tonic-clonic seizure, and only 35% have hemiclonic seizure; 1/3 of patients complain of the status epilepticus while 7% never have this feature; in patients with heterozygous *SCN1A* pathogenetic variants the disorder may occur up to 19 months (58). In most cases, DS is related to a genetic disorder, and in particular, the syndrome is linked by pathogenic variants in the sodium channel gene *SCN1A*. Other genes associated with DS include *SCN2A, SCN8A, SCN9A, SCN1B, PCDH19, GABRA1, GABRG2, STXBP1, HCN1, CHD2*, and *KCNA2* (4, 55, 59–62). DS linked to the *SCN1A* gene shows a phenotype that is characteristic of DS diagnosis, and the term *SCN1A*-related epilepsy has been proposed. The *SCN1A* gene encodes nine mammalian voltage-gated sodium channel alpha subunits, and their mutations are one of the most common causes of seizures reported in 70–80% of patients with DS (54). *SCN1A* mutations may result in an inhibition of GABAergic inhibitory interneurons, leading to excessive neuronal excitation. This interneural hypothesis appears to be the most plausible mechanism of DS. According to this hypothesis, *SCN1A* haploinsufficiency that results in a defective encoded protein channel NAV1.1 affects various parts of the brain, including the cortex, cerebellum, basal ganglia, and hypothalamus, causing clinical signs, such as epileptic seizures, ataxia, neuronal dysregulation, and sleep disorders (56). Non-*SCN1A* genes can manifest with a clinical expression similar to *SCN1A*-SD such that they are not easily distinguishable (54). Other authors have suggested that patients affected by DS linked to other genes, such as non-*SCN1A*, show a phenotype that is not well correlated with the classical phenotype of *SCN1A*-SD (55). The treatment of DS has been attempted using various drugs. In general, sodium channel-blocking drugs are not advisable because genetic dysfunction in DS involves sodium channels. A retrospective United States study was conducted to analyze the efficacy and tolerability of stiripentol (STP) in patients with DS (63). Eighty-two children with DS were divided into four groups: group A consisted of children treated with STP alone; group B, STB with clobazam (CLB); group C, STB with valproate (VPA); and group D, STB plus CLB, and VPA. Seizure frequency was reduced in 2/6 in group A, 28/35 in group B, 8/14 in group C, and 30/48 in group D. The main adverse effects were registered in 38 patients, mainly consisting of sedation and reduced appetite (63). A French cohort cross-sectional study was conducted in 54 patients of various ages ranging from 2.5 to 22 years using single therapy with STP, VPA, and CLB. The therapy was maintained for a long time by 96% of the cohort, since the frequency and severity of seizures were reduced. However, only a few patients achieved seizure freedom (64). Good results with the use of STP in DS were obtained also by Cho et al. (65) and Myers et al. (66). The most common adverse events were anorexia, weight loss, sedation, and behavioral changes (66).

## Genetic Epilepsy With Febrile Seizures Plus (GEFS+)

Genetic epilepsy with febrile seizures plus (GEFS+) is defined as a familial epilepsy syndrome in which affected individuals within a family typically have a variety of epilepsy phenotypes varying from simple and complex FS with a generally benign course to FS+ and various types of epileptic seizures, such as absence seizures, myoclonic seizures, atonic seizures, myoclonic-astatic epilepsy, and rarely severe epileptic encephalopathy (67). GEFS+ was described in a large Australian family by Scheffer and Berkovic (6) with an unusual concentration of individuals with FS+ and myoclonic seizures, FS+, atonic seizures, and FS+ and myoclonic-astatic epilepsy. The pattern of inheritance is primarily autosomal dominant; however, familial cases of likely *de novo* mutations and autosomal recessive inheritance have also been reported (6). GEFS+ is reported to be linked to mutations in the *SCN1A* (68), *SCN1A, SCN1B* and *GABRG2* gene mutations which are genes involved in the neuronal voltage-gated sodium channel (68–71). A clinical and genetic analysis of families with GEFS+ was performed in 409 patients by Zhang et al. (67). These authors obtained the following results extending the classical phenotype: focal seizures without preceding FS in 16/409 (4%), classic genetic generalized epilepsies 22/409 (5%), and afebrile GTCS 9/409 (2%). FS was the most frequent clinical sign in 178/409 (44%) patients, followed by FS+ 111/409 (27%). One-third 50/163 (31%) of the families had a pathogenic variant in a known GEFS+ gene. Moreover, the authors (67) proposed to adopt the term “genetic epilepsy with febrile seizures plus” rather than generalized epilepsy with febrile seizure plus. Recently, Jiang et al. (72) reported a patient with *CHRNA4* variant as a possible cause of paroxysmal kinesigenic dyskinesia and GEFS+. Among nine families with generalized epilepsy with FS+, Singh et al. (73) found 91 individuals with a history of seizures and 63 with seizures consistent with the diagnosis of GEFS+ syndrome. The epilepsy phenotype was of the type FS in 31, FS+ in 15, FS with other seizure types, such as atonic, myoclonic, absences, or complex partial in eight, and myoclonic-astatic epilepsy in nine individuals. In GEFS+ families, the FS+ feature is reported in ~20% of cases. The prognosis for both cognitive capacity and seizures is benign, as the stage of development in this disorder is generally not impaired, and generalized tonic-clonic seizures tend to persist over the age of 6 years with or without fever as a trigger (73).

### Genetics of Febrile Seizures (FSs)

A family history of epilepsy, including FS, occurs in 20–40% of children with FS (29). In families having one child with FS, an additional family member (first-degree relative) complained of FS in 10–46% of cases (29). The observation of a high (but not complete) concordance rate for FS among monozygotic twins compared with dizygotic twins indicates that genetic and non-genetic (or non-autosomal genetic) factors provide a relevant primary contribution. A common genetic component underlying the wide spectrum of FASE pathology has been assessed by advanced molecular analysis and association studies (3, 67, 74). However, specific extensive efforts have been addressed to improve the interpretation of causal genes and risk loci in two disorders of FASE group: FS and GEFS+, which vary among different clinical subsets and mutations. Up to date, a significant contribution in this field has been provided by Nakayama (75). In the review, nine genomic susceptibility traits linked to FS have been described, designated as from FEB1 to FEB9 located on chromosomes 8q13-q21, 19p13.3, 2q23-q24, 5q14-q15, 6q22-q24, 18p11, 21q22, 5q31.1-q33.1 and 3p24.2-p23; furthermore, mutations in the cholinergic receptor nicotinic alpha 4 subunit (*CHRNA4)* (72), in the voltage-gated sodium channel subunit genes (*SCN1A, SCN2A* and *SCN1B*), and in the GABA(A) receptor subunit genes (*GABRG2* and *GABRD*) (75) have been characterized in GEFS+.

## Febrile Infection-Related Epilepsy Syndrome (Fires)

FIRES is a distinctive entity that presents with fever and seizures. However, its clinical and genetic characteristics differ from the others disorders belonging to the FASE group. FIRES is a severe epileptic encephalopathy that occurs during childhood. The affected children in the acute phase manifest with recurrent FS and febrile status epilepticus, followed by a severe course with intractable seizures and residual intellectual disability. The disorder has been previously named fever-induced refractory epileptic encephalopathy in school-age children or fulminant inflammatory response epilepsy syndrome. FIRES is defined as a disorder that requires a prior febrile infection starting between 2 weeks and 24 h before the onset of refractory status epilepticus, with or without fever at the onset of status epilepticus (9, 10, 61, 76). The clinical course usually runs in three phases: in a normal child, there is a sudden onset of increased temperature, followed by an acute onset of recurrent seizures and refractory status epilepticus in the absence of fever with evidence of typical seizure type, and by severe drug-resistant epilepsy and neuropsychiatric involvement. The disorder is rare and has been estimated to have an incidence of one in 1,000,000 and a prevalence of one in 100,000 (77). The fever is initially high with a more or less duration of a few days. Seizures are initially focal, brief, and scanty, with rapid evolution within a few hours or days to status epilepticus. The EEG shows a diffuse slowing in the first phase to become multifocal, often involving temporal and frontal regions (61, 76). In seven patients with FIRES, Farias-Moeller et al. (78) disclosed three distinct features: a gradual increase in seizure burden, the presence of a recurrent extreme delta brush, and a typical seizure pattern. MRI is abnormal in 70% of cases with T2/FLAIR hypersignals located in the limbic and/or neocortical areas. Other areas, including the basal ganglia and peri-insular involvement, have also been involved (9, 10). T2/FLAIR hyperintense foci were found in the bilateral claustrum 10 days after status epilepticus onset (79). The etiology of FIRES is unknown although there is evidence of a possible neuro-infection or neuroinflammatory pathogenic role (80–82). A multicenter study conducted on 77 children with FIRES displayed a previous febrile infection reported in 96% of children. Brain autopsy performed in 13 patients showed gliosis in seven patients and leptomeningeal inflammatory infiltrates in one (9). Extensive cerebrospinal fluid examination, including cellular counts, polymerase chain reaction, and serologic studies, was inconclusive for a specific infectious agent in these children (9). Autoantibodies of known biomarkers for rheumatologic diseases as well intrathecally produced oligoclonal bands and antibodies against neuronal epitopes known to be associated with neurological disorders gave negative results (9). Recently, a case of FIRES-like condition due to autoimmune encephalopathy related to GABAAR antibody was reported in a 13-year-old girl (10). Hsieh et al. (81) reported impaired Toll-Like Receptor (TLR) responses and significantly lower cytokine profiles of TLR3, TLR4, TLR78, and TLR9 responses in children with FIRES, suggesting weakened phagocytosis and decreased T regulatory cell anomalies in affected children.

Different types of treatment for FIRES have been proposed. Anticonvulsant drugs have been shown to have poor efficacy and no effect on outcomes. In patients with FIRES, mostly in the chronic stage using cannabidiol treatment, Gofshteyn et al. (82) showed an improvement in seizure frequency and duration in six out of seven patients after 4 weeks (90%) and in 65% of the patients after 48 weeks. Nabbout et al. (61) reported an improvement in seven out of nine FIRES patients with a ketogenic diet within 4–6 days after the onset of treatment. Van Baalen et al. (77) have hypothesized that FIRES is an immune, but not an autoimmune disorder, and advised the use of GABAergic therapy at high doses and treatment with enteral or even parenteral ketogenic diet as the most promising type of treatment. In the study of Kramer et al. (9), no efficacious treatment was found in FIRES patients except intravenous immunoglobulin in two patients, ketogenic diet in one, and a prolonged cycle of barbiturates in one patient. Also, 9 patients (11.7%) died during the acute phase, 63 patients (93%) had refractory epilepsy, and only 12 of 68 (18%) surviving patients had a normal cognitive level (9). Based on FIRES immunologic mechanisms, Horino et al. (83) administered add-on intrathecal dexamethasone (IT-DEX) in six FIRES-affected children showing an improvement in seizure spreading and background activities on EEG monitoring, suggesting that IT-DEX treatment in FIRES children may shorten the duration of the critical stage of the disorder. Koh et al. (80) advised the early administration of a ketogenic diet and anakinra (an IL-1 receptor antagonist). FIRES is considered a subcategory of the “New-Onset Refractory Status Epilepticus” (NORSE) (84, 85). NORSE is defined as a clinical presentation characterized by new-onset refractory status epilepticus in a patient without active epilepsy and pre-existing relevant neurological disorders (acute strokes, brain masses, drug overdoses, and others) and without a clear acute or active structural, toxic, or metabolic cause) (85–87). The relationship between FIRES and NORSE and their boundaries remains to be established as referred by Bhatia and De Jesus (88). Patients with NORSE may or may not be associated with fever before the start of refractory status epilepticus. Nausch et al. (89) who in a retrospective comparison of 18 adults with NORSE vs. 48 children with FIRES revealed more differences than similarities. To date, many doubts remain regarding etiologic factors, diagnostic criteria, therapeutic options, and other questions, including the limits of NORSE disorders (84, 90). Within the FASE group, FIRES is a rare disorder; however, it is extremely severe with mostly irreversible cerebral damage.

## Conclusions

FASE defines a distinct group of entities with fever and seizures as the main features in which underlying common clinical, immunological and genetic pathways have been recognized. The pathogenesis of these disorders may be multifactorial, with variable clinical expression from simple and benign FS to dramatic evolution, as in FIRES. With the exception of simplex and complex FS, most of these disorders are associated with systemic complications and severe outcome. Various types of treatment exist for each of the disorders belonging to the FASE group, but in some of them any type of therapeutic treatment may still result inconclusive. Despite much progress made till now in the clinical and genetic diagnosis of disorders belonged to FASE, more research efforts, particularly from translational studies should be oriented to drug-target therapy.

## Author Contributions

PP, XGP, and EP drafted and redrafted the present manuscript. XGP and JKF helped to analyse the genetic data of the literature review. RF and MR contributed to the clinical understanding of the literature findings and revised the manuscript. SDM reviewed and edited the draft. All authors read and approved the final manuscript.

## Conflict of Interest

The authors declare that the research was conducted in the absence of any commercial or financial relationships that could be construed as a potential conflict of interest.

## Publisher's Note

All claims expressed in this article are solely those of the authors and do not necessarily represent those of their affiliated organizations, or those of the publisher, the editors and the reviewers. Any product that may be evaluated in this article, or claim that may be made by its manufacturer, is not guaranteed or endorsed by the publisher.
